# To treat or not to treat? Oncologists’ perceptions and experiences regarding overtreatment in end stage cancer patients

**DOI:** 10.3389/fmed.2025.1607479

**Published:** 2025-07-10

**Authors:** Saritte Perlman, Aviad Raz, Pesach Shvartzman, Raphael Catane, Tamar Freud, Moriah E. Ellen

**Affiliations:** ^1^Department of Health Policy and Management, Ben Gurion University of the Negev, Beersheba, Israel; ^2^Israel Implementation Science and Policy Engagement Center, Ben Gurion University of the Negev, Beersheba, Israel; ^3^Department of Family Medicine, Siaal Research Center for Family Medicine and Primary Care, Ben Gurion University of the Negev, Beersheba, Israel; ^4^Shaare Zedek Medical Center, Jerusalem, Israel; ^5^Institute for Health Policy, Management and Evaluation, University of Toronto, Toronto, ON, Canada

**Keywords:** overuse, end-of-life, oncology, qualitative, palliative care

## Abstract

**Introduction:**

Aggressive care at end-of-life can harm quality of life without significantly improving survival. Despite best practice guidelines, research shows that oncologists continue to provide too much treatment to patients, especially at the end-of-life. Understanding the perceptions of oncologists regarding unnecessary care toward end-of-life can inform interventions and mitigate overuse. This study aimed to understand the perceptions and experiences of oncologists regarding why overuse of services is occurring for cancer patients at the end-of-life and elucidate factors which impede the implementation of best practices at the end-of-life in cancer.

**Methods:**

In-depth, semi-structured interviews were conducted with oncologists in Israel. The interview guide was based on the Theoretical Domains Framework to identify beliefs about practices in caring for patients at the end-of-life and transitioning to palliative care. Interviews were audio-recorded, transcribed, coded, and thematically analyzed.

**Results:**

Participants identified six major barriers and 12 major facilitators to reducing overuse at end-of-life. Barriers included patients seeking second opinions, patient and family fragility, pressure and demands from patients and families, a culture of valuing extending life, time constructs, and physicians’ emotional regulation. Physicians reduce overuse by relying on experience, communication and relationship building skills, taking ownership over their roles, confidence in their abilities, belief and recognition of the importance of appropriate care, involving families and other healthcare professionals and easing into the process. Oncologist opinions vary based on role and geographical area of practice.

**Conclusion:**

Physicians can influence the rate of overuse as they guide patients at end-of-life. Findings can be utilized to help the health system in Israel reduce the overuse of unnecessary services at the end-of-life for cancer. Interventions such as palliative care referrals, multidisciplinary teams, and educational initiatives can help minimize overuse and improve quality of life for patients in their final days. Future research should incorporate views and perspectives of other stakeholders.

## 1 Introduction

Provision of high quality health care services has increasingly become a point of focus across global health systems, especially in the context of limited resources (1, 2). Quality issues on the spectrum of underuse, misuse and overuse occur when services are provided ineffectively or inefficiently to patients who may not benefit from them (3).

Globally, increasing attention is being paid to overuse (4), referring to “care that can lead to harm and consumes resources without adding value to patients” (5). Various studies have documented that somewhere between 20% and 33% of patients receive unnecessary, ineffective, or potentially harmful care (6–9), characterized as “a disservice to patients who are subjected to ongoing and likely uncomfortable conditions with no benefit” (10). Overuse of health services has been found in most demographic groups and disease states (4, 11, 12) and the broader literature has identified a complex array of variable factors at the patient, provider and system levels (4).

One in every six deaths worldwide is due to cancer, making it the second leading cause of death globally (13). Currently, approximately 19 million new cases of cancer are diagnosed annually, costing an estimated $1.16 trillion (14). While best practice guidelines and “do not do” recommendations often target oncology services, overuse persists (12, 15).

The United States National Cancer Board states that poor quality care occurs when “practices of known effectiveness are being underutilized, practices of known ineffectiveness are being over utilized, and when services of equivocal effectiveness are being utilized in accordance with provider rather than patient preferences” (16). Due to anticancer treatments often being invasive to patients and costly to the system, there is reason to believe that they should be prioritized when trying to reduce inefficient use (17).

A systematic review of studies concluded that overuse rates within oncology can be very high. For example, 49% of services within breast cancer, 32% within prostate cancer, 56% of diagnostic tests, and 71% of imaging tests in oncology could be classified as unnecessary (18). Though there is a lack of information on the extent of this phenomenon in Israel, it can be assumed that data are not significantly different from those published in other Western countries.

Israel is unique in its ethnic makeup, being the only country with a majority Jewish population. Certain sub-populations of Jews are at particularly high risk for certain cancers, such as colorectal, ovarian, pancreatic, stomach and breast, the distribution of which would be unique to Israel (19–21). Thus, Israel should seek the specific factors which contribute to the overuse of cancer services applicable to its unique context.

Cancer has been the leading cause of death in Israel since 1999, and the oncology specialty has grown with this increasing burden. The National Cancer Registry has documented increasing cancer prevalence due to population growth and case accumulation. Health technologies for cancer occupy about one-third of Israel’s yearly budget for all health services provided by the National Insurance Law of 1995, which provides all Israeli citizens with a basket of health services (22–24). An Israeli study analyzing cancer costs within Maccabi Health Services identified that there was a wide variation of cost-effectiveness and budget impact of various cancer drugs. They concluded that the challenge of distributing finite resources will only increase and it is vital to consider these issues in the Israeli cancer context (25, 26). Another study surveying primary care physicians and oncologists in Israel found that cancer treatments are regarded as being of higher value, which has been colloquially referred to as a “cancer premium “(27). Regarding overuse of cancer services, an Israeli study found that 18.8% of oncology patients received chemotherapy within 2 weeks before death (28).

Research has identified potential areas of overuse and aggressive care toward the end-of-life including utilization of chemotherapy and radiotherapy very near death; underuse of or late referral to hospice services indicating the overuse of aggressive care; and high rates of emergency department usage, hospitalization, and intensive care stays close to death (29). Overall, “quality care does not reach enough people (at the end-of-life),” and this imposes a significant burden on patients, their families, and the healthcare system (30). Studies have shown that early referral to palliative care and refusing aggressive care can lead to higher quality of life and longer survival (17, 31, 32).

Bringing about change in this area should utilize a multipronged approach, including both top-down efforts, such as national policy reforms, institutional protocols, and updated clinical guidelines, and bottom-up approaches such as oncologist-led communication training, peer mentorship, and team-based palliative care integration. Engaging in conversations with a patient concerning end-of-life and associated options is correlated with the patient undergoing less aggressive care near death, reduced costs to the system, improved quality of life, as well as higher patient and caregiver satisfaction (33, 34). Many variables impede oncologists’ ability to discuss these issues and effectively guide a patient including the fear of destroying hope, damaging the therapeutic relationship, or triggering death anxiety in the patient (35).

A Canadian study examined strategies and barriers among Canadian oncologists for effective communication about end-of-life with patients (36). The barriers found included difficulty in treating and palliating patients concurrently, discomfort surrounding death, ambiguity surrounding the responsibility to lead these discussions among treating staff, excessive focus on healing, and lack of experience and good mentorship. Barriers were also found on the part of patients and their families, such as unwillingness to conduct an end-of-life conversation, language barriers and patient’s young age. Organizational factors included stigma surrounding palliative care, lack of a uniform protocol for end-of-life care, and lack of tools and training for such communication. Meanwhile, a recent study in Israel found that current oncology training programs lack a focus on the emotional component of end-of-life conversations with patients which has negative consequences for physicians and patients. The researchers concluded that further work should be invested in improving this capability among oncologists (35).

Oncologists are the leading figure among the members of multidisciplinary oncology teams, due to their specific role in the physician-patient relationship and as the primary source of ordering various tests, treatments and procedures. Understanding their unique attitudes and experiences may set the foundation to provide intervention opportunities to address overuse (37).

Related research by our team explored the perceptions of different oncologist groups in Israel surrounding overuse across the spectrum of oncology services (38). Participants identified causes of overuse at the patient-level, including “well-informed” and “demanding” patients; the physician-level, including the need to pacify patients, and lack of confidence, time, and skills; and the system-level, including ease of access and lack of alignment and coordination. Participants also identified facilitators to reduce overuse and suggested improvements such as dialoguing with patients, building trust and patient-provider relationships, and the further reduction of overuse through teamwork. On a systems level, participants felt that the regulation of services minimizes overuse, and more improvements can be made through education, and bottom-up solutions. These findings from our previous study provided the foundation to ask further questions including exploring overuse at the end-of-life in cancer care. Although many oncologists are aware of this issue, as well as the best practice guidelines which underscore the importance, unreasonable variations continue to exist in referral to palliative and hospice care (38, 39). The objectives of this study were to understand the perceptions and experiences of oncologists regarding why overuse of services occurs for cancer patients at the end-of-life and elucidate physician level factors which impede or help facilitate the implementation of best practices at the end-of-life in cancer.

## 2 Materials and methods

In-depth telephone interviews were conducted with oncologists in Israel using A semi-structured interview guide. This approach is well-suited where little data is available, as participants can respond freely and the interviewer can further explore relevant issues (40, 41) as is the case regarding oncologists’ knowledge and perceptions on overuse of services at the end-of-life.

### 2.1 Sample selection and recruitment

Interviews were conducted with hospital-based oncologists from various medical centers across the country. Interviews were conducted with three groups of oncologists to best reflect the perspectives of participants at different levels of seniority: current or former department heads, oncology specialists, training oncologists (residents and fellows). Recruitment was done by contacting oncologists using email addresses published on the medical centers’ websites and utilized snowball sampling, whereby interview participants were asked to identify other potential participants after completing the interview. Lastly, personal networks of the research team were used to recruit additional interviewees. All participants signed consent forms after being informed that their opinions remain anonymous, their participation was completely voluntary and that they could withdraw from the study at any time.

### 2.2 Data collection and analysis

This study used the Theoretical Domains Framework (TDF), which was developed to identify determinants of behavior, to understand why healthcare practitioners are ordering unnecessary tests, treatments and procedures toward the end-of-life. The TDF is a comprehensive framework focusing on determinants of healthcare professional behavior that synthesized theoretical constructs and psychological theories into 14 domains focusing on cognitive affective, social, and environmental factors (40, 42–44). The framework has been operationalized in numerous healthcare settings to understand clinical behaviors such as blood transfusions (45), oncology treatment (46), antimicrobial stewardship (47), diabetic retinopathy screening (48), prescription errors (49), and hand washing (50).

The interview guide was developed based on the TDF and consisted of an open question for each theoretical domain followed by a series of prompts. To maximize the natural flow of conversation, flexibility in the order in which domains were covered was used. Interviews were digitally recorded and transcribed by a professional transcription firm. Atlas.ti software (ATLAS.ti Scientific Software Development GmbH, Berlin), was used to code, store, and organize data. The research team included members trained in medicine, pharmaceutical science, health system research, clinical research, and qualitative methods. Senior team members brought direct clinical experience as oncologists or in palliative care, while others had prior involvement in oncology-related health services and implementation science research. This diversity of expertise contributed to a nuanced interpretation of the data. We recognize that our disciplinary backgrounds and professional experiences could influence data interpretation. To mitigate potential biases, we employed strategies to enhance reflexivity throughout the study.

Data was analyzed simultaneously to data collection. Field notes and memoing were used to document impressions and analytic decisions. The first 7 (23%) interviews were double coded by two researchers independently, discrepancies were discussed, and coding guidelines refined to develop a codebook for content analysis. The remaining interviews were analyzed by one researcher using the collaboratively developed codebook. Analysis was inductive and involved line-by-line coding, with codes and categories developed from participants’ responses. Focused and theoretical coding were used to develop core themes.

Thematic saturation was assessed iteratively during the data collection process. After approximately 10 interviews per group, the coding team noted that new interviews no longer introduced novel themes or altered the coding structure. At this point, additional data largely confirmed and enriched existing themes rather than expanding them. This approach aligns with established qualitative research practices, which prioritize depth and diversity of perspectives over numerical thresholds for sample size.

## 3 Results

Seventy-eight oncologists from across the country were approached; 33 agreed to participate, two were lost to follow up and one subsequently withdrew from the study. Interviews duration ranged 15–70 min (mean 34 min). Twelve participants were current or former department heads, 10 were oncologist specialists, eight were training oncologists. There was even participation between genders (14 males, 16 females). Participants studied medicine in six countries and completed their oncology training in four countries (29 participants completed at least part of their oncology training in Israel). Oncologists worked at 15 different hospitals, and in the community; 10 (33%) participants worked in the southern region, eight in the central region (26%), eight (26%) in the northern region, and four in Jerusalem (13%).

### 3.1 Perceived barriers to reducing overuse

Participants identified six major barriers to the implementation of best practices in end-of-life care in cancer and causes of overuse. Themes spanned four TDF domains: social/professional role and identity, belief in consequences, environmental context and resources, and emotion (Table 1).

**TABLE 1:** Major barriers identified according to respective Theoretical Domains Framework (TDF) domains.

TDF domain	Theme	Details
Social/professional role and identity	Patients seek second opinions	Patients seeking consults with other oncologists until they receive the sought-after response or treatment.
Belief in consequences	Patient and family fragility	Fear of potential negative emotional/psychological side effects for patients and families.
Environmental context and resources	Pressure and demands from family members	Demanding nature of family.
	Pressure and demands from patients	Demanding nature of patients.
	Culture of valuing life	Cultural value of extending life at all costs.
	Time constructs	Time demands and constraints.
Emotion	Physician emotional regulation	Challenging for physician to stop active treatment, conduct end-of-life conversations and transition patients to supportive care.

#### 3.1.1 Social/professional role and identity

##### 3.1.1.1 Patients seek second opinions

Some patients seek additional opinions after end-of-life conversations during which their oncologists recommended ending active treatment, “some also do a round of second opinions between oncologists and surgeons and all sorts of people, streams of alternative medicine” (P-15). Some patients may be successful, “if you don’t do it, then they will go to another doctor who will do it. That is, they will find the person who will agree to do what they want” (P-2). Other times the consulted oncologist will provide an opinion that is in line with the original decision, “if the doctor is decisive, they will say “this is how it is”” (P-30).

#### 3.1.2 Belief in consequences

##### 3.1.2.1 Patient and family fragility

Oncology patients and their families experience physical, psychological, and financial burdens and some oncologists cited that they worry about the emotional impact of stopping treatment, “there are kinds of patients and families who when told “there are no more treatments,” it only makes them fade faster” (P-3). Participants believing that there may be a non-medical negative impact on the patients can lead to a reluctance to stop treatment, “it is difficult for them to accept that there is no more cure. And it doesn’t matter how many things you have already gone through with them, once you say there is nothing more to give, they think you are throwing them away. Statements like that can be very harsh. But that is the patient’s experience. I have nothing to argue with this experience. That’s how they feel” (P-9).

#### 3.1.3 Environmental context and resources

##### 3.1.3.1 Pressure and demands from family members

Participants recognized that families need to feel that they have done all that is possible, “it is very noticeable, for example in the cases of parents of young people. They want to feel that they have done everything to the end” (P-8). Some participants referred to family members as sources of pressure for patients, pushing them to ask for unnecessary treatments, or otherwise asking on their behalf, “there are situations where family members are not willing to give up and the patient is. Then they will bring the patient in for another conversation and then maybe (for) the next conversation (the patient) will come with another family member and then (for) the next conversation they will come with another son. It happens in those big families that have a lot of children, and a lot are involved in the patient’s life. And you don’t always hear what (the patient) wants” (P-25).

##### 3.1.3.2 Pressure and demands from patients

Participants mentioned patients’ desires for continued treatment, regardless of their decreased function and oncologist’s opinions, “there are patients who want to receive treatment at any cost” (P-4). Patients’ insistence on continued therapies can lead to unnecessary care, “sometimes you make decisions that are dictated to you, sometimes because of the patients’ desires” (P-10). Patients’ demands may come from their desire to fight until the end, “they feel that this way they are fighting to the end” (P-13). The pressure that is put on oncologists can be overwhelming, “they somehow demand and demand and demand from us, and we really feel (like we are in a) pretty unpleasant situation” (P-15). Some oncologists end up giving in, “so I provide (it)” (P-2).

##### 3.1.3.3 Culture of valuing life

Participants discussed the culture of valuing extending life above all, and the associated challenges, “there is a certain situation here that is quite unique to the country, some sort of value of fighting for life in a way that (goes) above and beyond” (P-18). Many participants mentioned how the culture in Israel compares in contrast to the approach and acceptance of end-of-life in other countries, “I always say, “In Israel I learnt how to treat, in the United States I learnt not to treat, and the truth is somewhere in the middle”” (P-17).

##### 3.1.3.4 Time constructs

Most participants spoke about the lack of time to conduct necessary conversations, “there is never enough time, you know” (P-27). Lack of time comes at a cost to the oncologists, “at the end of the day, and (often), the time comes at our own expense. (We) don’t manage to drink, eat, go to the bathroom within the time and get delayed at work. All kinds, we get to the edge of physiology sometimes” (P-15). However, conversations are critical and time-sensitive, so oncologists adapt accordingly, “if it’s necessary to do it–I do it, and then I have a delay in patients” (P-13).

#### 3.1.4 Emotion

##### 3.1.4.1 Physician emotional regulation

Participants cited the emotional strength necessary for managing end-of-life conversations and dealing with death as oncologists, “sometimes it is really hard” (P-3), while many mentioned that it is always hard. Participants expressed the weight on them personally, particularly when patients are younger, have a late-stage diagnosis with expected quick course of disease, and when they have an established relationship with the patient. One participant explained, “realizing and informing the patient that the treatment has failed. That’s what’s hard. This is not a break. The hard part is failure, and you know what’s going to happen” (P-2). Some participants mentioned coping mechanisms for managing the emotional burden, “I did a lot of meditation workshops and lot of spiritual things and conversations and workshops, and I also went to private psychologists” (P-15).

Participants across all groups mentioned the demands of patients and their families as a significant barrier to providing appropriate care. Regarding the emotional toll it takes on oncologists, mentions decreased with level of seniority. Department heads spoke the most about cultural factors and time constraints. Oncologists who practiced in major cities cited cultural barriers much more than oncologists who work in peripheral cities.

### 3.2 Perceived facilitator to reducing overuse

Participants cited many physician-level factors that can facilitate the implementation of best practices at end-of-life. Major factors spanned 6 of the TDF domains, illustrating the complex, multifaceted nature of the issue (Table 2). In many cases, a lack of cited facilitators was identified as a potential barrier.

**TABLE 2:** Major facilitators identified according to respective Theoretical Domains Framework (TDF) domains.

TDF domain	Theme	Details
Knowledge	Professional and clinical experience	Personal and clinical experience informing knowledge and understanding of end-of-life conversations, transitions and care.
Skills	Communication	Explaining clearly, honestly and openly, and listening to patient’s needs.
	Relationship building	Building trust and relationships with patients, and families.
Social/professional role and identity	Ownership of role and responsibility	Oncologists’ ownership of responsibility to conduct end-of-life conversations and transition patients to supportive care.
Memory, attention, and decision making	Do not forget about transitioning care	Do not forget to conduct end-of-life conversations once decision to stop active treatment has been reached.
Environmental context and resources	Familial involvement	Involvement of family as support system for patients at end-of-life.
Social Influences	Consultations with other healthcare professionals	Team and individual consults with colleagues.

#### 3.2.1 Knowledge

##### 3.2.1.1 Professional and clinical experience

Participants repeatedly mentioned the significance of personal, professional and clinical experience when identifying and deciding to stop providing active care. Managing overuse at end-of-life improves over time, “we will start with the medical knowledge that accumulates over the years” (P-2). One participant noted the importance of extensive knowledge, “for these sorts of decisions you need to know the field” (P-10), while another noted, “therefore it is a matter of experience” (P-14). More experience can also ease the process of letting go, “the more experience you have in the matter the more you understand when to let go” (P-5).

#### 3.2.2 Skills

##### 3.2.2.1 Communication

Participants’ competence and ability to clearly communicate with patients and family members was cited as particularly important when conducting conversations about protocol cessation and transition to palliative or supportive care. Basic communication skills were implied, “first of all communication skills” (P-18) including “open dialogue with the patient” (P-16). Interpersonal skills served as a facilitator when the oncologist’s skill level was high, and a barrier when their skill level was lower. Listening to patients and their families was seen as highly important, “(being) attentive to the opinion of others and attentive to the feelings of the patient and their family” (P-12) and “let (ting) the patient express themselves, let them ask questions”’ (P-18). Another participant shared, “you need good communication with the patient, with the family, empathy, sensitivity.” (P-6).

##### 3.2.2.2 Relationship building

Existing relationships of trust between patients and their providers were perceived as a foundation to be relied upon, “first of all, the trust that has been created between the doctor and the family and the patient” (P-23). As one participant explained, “(it) requires a lot. Good relationships with the patient and a lot of investment in the relationship with the patient. To truly get to the right place in terms of mental (health) and professional oncology treatment for the patient” (P-25). Oncologists who were not able to successfully build or foster relationships with patients and their families were faced with a barrier when trying to minimize overuse, “if you don’t build it during the period of the disease, from the beginning, slowly, then it does not work out well at the end either” (P-1).

#### 3.2.3 Social/professional role and identity

##### 3.2.3.1 Oncologist ownership of role and responsibility

The perception among most of the participants was that oncologists assume responsibility for identifying when active treatment becomes unnecessary and inefficient, “you believe it’s part of your job as an oncologist, as a treating physician” (P-6). A few participants reluctantly added that the ultimate decision is the patient’s and if the patient wants to continue active treatment it is not within an oncologist’s professional boundaries to deny them, “the patient has a voice, and the final decision is of course theirs” (P-12).

#### 3.2.4 Memory, attention, and decision making

##### 3.2.4.1 Do not forget about transitioning care

Participants explained that once a patient has been identified as approaching end-of-life at a stage where additional active treatment will not add value, they do not forget to speak to their patients about transitioning care, “if you have to have the conversation, then you have to have the conversation” (P-17). Participants added that these conversations are not delayed and prioritized so transition can begin, “we don’t forget because it is my duty not to do harm” (P-31) and “I think that the majority don’t stretch it out” (P-20).

#### 3.2.5 Environmental context and resources

##### 3.2.5.1 Familial involvement

Participants recognized the invaluable nature and importance of patients’ family and friends as support networks and taking their opinions and needs into consideration, “usually for a (n end-of-life) conversation, for such conversations, the patient comes with their wife or children. Most oncology patients don’t come alone” (P-14). As a facilitator to the process, family members may be able to better understand the situation, “if a family cooperates and you can talk to them and try to convince them, and they themselves actually understand, maybe look at it a little from the outside, understand that the disease will not be affected by treatment and come to terms with it more” (P-16).

#### 3.2.6 Social influences

##### 3.2.6.1 Consultations with other healthcare professionals

Participants discussed their patients with other oncologists and teams, “you can always consult (with) anyone” (P-21). Consultation was particularly important for training oncologists as part of the mentorship from more senior oncologists, “you can always consult with the senior (doctors) on how to (conduct) these types of conversations” (P-13). Consultation with the entire team or individual team members provided participants with the support and reassurance necessary to ceasing active care, “team discussions, a whole team decision, it’s not my decision” (P-19). One participant specified, “I think it’s very important to talk to- I, for example, talk to my friends here, doctors, with the oncologists or the supportive care unit, and share my challenges, and then I hear what they have to say” (P-18), another participant added, “I always prefer to approach the patient (being able to) say “I talked to colleagues, I consulted.” It provides some support” (P-27).

Between the different oncologist groups, departments heads, and oncologist specialists mentioned other healthcare professionals, as a support resource and as a social influence, much more than training oncologists. The social influence of consulting with other healthcare professionals was mentioned most by the oncologist specialists and much less by training oncologists. Department heads mentioned the helpful involvement of families the least. Overall, department heads and oncologist specialists were more like one another, while training oncologists had different perspectives. Fewer overall differences were found between groups based on geographical location of practice however, oncologists from Jerusalem cited the benefit of personal and clinical experience significantly less than their counterparts.

## 4 Discussion

This study examined Israeli oncologists’ perceptions of overuse in oncology services at end-of-life in order to elucidate physician-level factors which impede the implementation of best practices at the end-of-life in cancer. Difference in responses between groups in this study reflect the effect of professional roles and responsibilities, levels of training and education around palliative care. The range of factors cited sheds light on the complexity of minimizing overuse, but the many facilitating factors demonstrate a foundation for change. A multipronged approach is required to mitigate these barriers while building upon facilitators.

The findings of this study concur with previous research in the fields of end-of-life, behavior change and overuse in general, highlighting lack of time, need for multidisciplinary professional collaboration, importance of effective communication, and emotional challenges (36, 38, 51–54). Furthermore, this study fits with the previous finding that oncologists’ perception of patients’ sensitivity and fear of transitioning to palliative care can potentially lead to over-medicalized end-of-life (55). While previous research reported that beliefs about capabilities can be a barrier for healthcare professionals, participants in this study cited confidence in their abilities (53). This difference may be attributed to the fact that this study only included oncologists and be a cumulative effect of the culture of confidence among both oncologists and Israelis (52).

A recent study conducted in the Netherlands found that consulting oncologists are generally cautious when approached for second opinions regarding prognosis, and often support the initial opinion (56). Their study did not discuss receiving treatment from a consulting oncologist, whereas participants in our study referred to seeking second opinions as well as receiving treatment from other oncologists. However, their study did not discuss receiving anti-cancer treatment from a consulting oncologist. In contrast, participants in this study, explicitly mentioned the negative effect of patients seeking second opinions and the culture of valuing extending life. In a previous study conducted in Israel, patients “shopping around” seeking their desired opinion was also cited as a barrier to reducing overuse (38). Israeli patients’ health seeking behavior can be contextualized as an additional cultural element to be addressed delicately.

Israel’s approach to extending life is documented in the literature surrounding the bioethical nature of end-of-life policies and laws which are influenced by Jewish law’s prioritization of sanctity of life and restriction on any life-shortening interventions (57, 58). In this way, religion and tradition are deeply rooted in the society, even though not all Israelis are either Jewish or religious. As such, the culture is one of rigorous and intensive medical treatment and society thinks positively about using medical interventions to alleviate pain and suffering (59). Notably, oncologists from more urban centers and department heads cited cultural barriers more than their colleagues, perhaps due to related to treating more homogeneous populations and/or smaller and more specialized caseloads (60). Addressing this cultural barrier requires nuance and understanding, interventions should build on trust and cultural understanding.

Furthermore, the “Dying Patient Act” enacted in 2005 established guidelines that both raised dilemmas and challenges for practitioners, while pushing end-of-life issues to the forefront. This law establishes clear guidelines for healthcare providers regarding the treatment of dying patients, emphasizing the importance of respecting patients’ wishes while also addressing cultural and religious considerations that oppose practices like euthanasia and physician-assisted suicide (61, 62). The act mandates palliative care as a right and outlines the responsibilities of healthcare professionals, including the appointment of a senior physician to oversee care decisions but it has been critiqued for its restrictive nature, which can lead to under-regulation and ambiguity in practice (63, 64). While the “Dying Patient Act” aims to promote patient dignity and autonomy, its implementation reveals complexities that necessitate ongoing dialog and training for healthcare providers (61, 65). Unlike jurisdictions such as the Netherlands, Canada, or select U.S. states where active euthanasia or physician-assisted suicide is permitted under strict conditions, Israel prohibits both, reflecting the halachic principle of the sanctity of life. The law permits passive euthanasia, such as withholding or withdrawing treatment, but only under tightly regulated circumstances. In contrast to secular Western democracies, where patient autonomy is often the guiding principle, the “Dying Patient Act” reflects a physician-duty model, embedding religious and ethical constraints within a technocratic legal framework (66). The law represents a cultural compromise, balancing modern medical ethics with traditional religious values, and stands apart from more secular, autonomy-driven approaches elsewhere. The findings of this study should be interpreted considering these national and cultural particularities, and the transferability of results to more autonomy-focused systems should be considered with adaptation to local context.

Oncologists in this study expressed how emotionally challenging it can be to stop providing their patients anticancer treatment. Oncologists want to minimize the overuse, take action to ensure their patients are receiving the appropriate care by not delaying end-of-life conversations and by providing more appropriate supportive care. Framing utilization of palliative care as a positive action that can be taken by oncologists may help increase earlier integration of end-of-life care and minimize overuse. Previous research in other Western countries found that some healthcare professionals only considered palliative care after stopping all curative treatment as they believe the term is only applicable to terminal patients (55, 67). However, palliative care has been defined as “medical care focused on the relief of suffering and support for the best possible quality of life for patients facing serious, life-threatening illness and their families” and can be provided in tandem to curative treatment (68). When referred to as “supportive care” instead, research found that there was a significant increase in the number of patients referred for treatment, and shorter times before supportive care consultations (69). This demonstrates the importance of semantics and framing for both patient and provider, but also a potential gap in oncologists’ knowledge and understanding of palliative care.

Research has found that healthcare professionals do not always have a clear conceptual understanding of palliative care (53, 55, 70). Many large-scale educational interventions have been developed and implemented targeted to medical students, and healthcare providers in general, or specialized educational initiatives for palliative care education and publication of educational resources (71–73). While the awareness of overuse has been increasing in Israel and include best practice guidelines, further distinction between overuse and appropriate care may be necessary. Existing interventions should be prioritized for local adaptation and implementation. Engaging medical-oncological societies in a bottom-up approach may assist in starting the discussion with healthcare providers in oncology regarding overuse in general and at end-of-life.

Recent research in Israel has found that psycho-social-spiritual interventions are associated with reduced aggressive end-of-life measures and extending the time between active treatment and death. The study authors recommend building structured two-stage “palliative conversations” by having patients speak with their oncologist and subsequently with a psycho-social-spiritual professional, each of the respective professional providing support in a different and complementary manner (74). Strategies to address pressure and demands from patients and families should build on the skills-related themes of communication and relationship building and involvement of other healthcare professionals. Strategies should focus on shared decision-making and communication interventions that enable physicians to confidently and appropriately discuss treatment options with patients and for patients to feel informed and comfortable in the decision (75, 76). Improving multidisciplinary healthcare professional involvement such as partnering with oncology nurse practitioners and palliative care teams can be beneficial in addressing the needs of patients and families and ultimately easing transitions at end-of-life (54, 77). As study participants did not discuss the availability of palliative care teams and practitioners, ability to refer to home or hospital hospice care, waiting lists or other non-treatment options. These potential tangible barriers may need to be addressed with health care services planning and in addition to attitudinal changes.

While the interventions proposed in this discussion are applicable to the barriers identified, they should be monitored and evaluated to assess their effectiveness in the Israeli context. Future research should also aim to quantify the nature of overuse in Israel across specialties and services to determine an accurate representation of the issue and should explore the perceptions and perspectives of overuse of other healthcare providers, families and patients.

### 4.1 Strengths and limitations

This study’s primary strength and unique contribution is that it is the first of its kind in Israel focusing on oncologist-perceived factors of overuse at end-of-life. Within Israel, our sample was diverse, featuring participants from different hospitals, regions and at different stages in their careers. Using the TDF framework for this study provides a theoretical basis for future implementation studies.

This study is not without its limitations. Participants that agreed to partake may have contributed to selection bias as they were already interested in the topic. This was mitigated where possible by combining recruitment methods and not relying solely on snowball sampling. Due to the context and culture-specific nature of our findings in this single-country focus, wider generalizability and applicability to other contexts will require local adaptation. Additionally, due to the COVID-19 pandemic, interviews took over a year to complete, follow-up was lost with some oncologists and regaining contact during the acute months of the pandemic was very challenging. As data collection was delayed and the response rate decreased, the entire study was delayed accordingly. Changes in the provision of care during the pandemic may also have had an indirect effect in the opinions of training oncologists who already have less clinical experiences.

### 4.2 Implications and recommendations for policy, practice and research

Overuse in any field of medicine, specifically in oncology, is extremely costly to the healthcare system and can cause harm, reduce quality of life, while not adding benefit. Results of this study demonstrate the importance of addressing the physician-level factors to encourage behavior change. Palliative care is an integral component of quality care for patients at the end-of-life, and an important system-level support for oncologists. Results of this study highlight the gaps between providing patients with curative or supportive care and inconsistent utilization of palliative care services in some practice settings. Current opportunities for palliative care education for all should be appraised by health system leaders and hospital and unit-based leadership should promote and encourage utilization of palliative care services as part of normal patient protocol, starting with education. Health system leaders should consider embedding mandatory palliative care consults within standard oncology pathways, particularly for patients with metastatic disease and limited prognosis or early palliative care consultations prior to initiating aggressive treatments in late-stage cancer patients. Furthermore, clearer clinical protocols and more precise interpretation of the “Dying Patient Act” are needed to alleviate legal ambiguity. Internationally, the interdisciplinary nature of palliative care has been integrated into oncology clinics including specialized nurses and oncology nurse practitioners, psychologists, social workers, pharmacists, and other allied health professionals each contributing unique expertise while working together in a cohesive manner to address patient needs (77, 78). A similar model should be assessed and considered in Israel. Additionally, regular audits of end-of-life care patterns and improved enforcement of existing guidelines may help reduce variation and overtreatment. Given the financial strain of continued non-beneficial treatment, policymakers should also consider integrating cost-effectiveness thresholds or value-based coverage policies into oncology reimbursement structures.

## 5 Conclusion

As health systems continue to pay increasing attention to providing high quality care and services to patients, identifying how best to minimize overtreatment is critical especially in clinical situations such as end-of-life care in oncology. Our work suggests that understanding oncologist perceptions can assist policymakers and decision makers in developing appropriate interventions to address the overuse of unnecessary cancer treatments at end-of-life by using a multipronged approach. If implemented appropriately, interventions could result in positive behavior changes by building on and reinforcing existing facilitators and by addressing barriers.

## Data Availability

The datasets presented in this article are not readily available. Interviews and coded data will be made available upon reasonable request. Requests to access the datasets should be directed to MEE, moriah.ellen@gmail.com.
